# ADHD in adulthood: Why should we not give up on getting the diagnosis right

**DOI:** 10.1192/j.eurpsy.2025.267

**Published:** 2025-08-26

**Authors:** J. S. Kooij

**Affiliations:** 1 Psychiatry, Amsterdam UMC/VUMc, Amsterdam; 2 Adult ADHD, PsyQ, the Hague, Netherlands

## Abstract

**Abstract:**

ADHD in adulthood is still an orphan diagnosis in many countries in Europe. This is due to lack of knowledge in the education of psychiatrists in adult psychiatry about the research findings on adult ADHD of the past 30 years. Why is this important?

ADHD is prevalent in about 3-5% of adults in the general population, but in at least 20% of patients in psychiatry, whether anxiety, depression, bipolar disorder, substance abuse disorders and many more. In adults with borderline personality disorder, ADHD symptoms in childhood precede the development of this personality disorder later in life even in the majority of cases. Not recognising and treating ADHD in adulthood means that this condition impacts the treatment outcomes of any other psychiatric comorbidity due to impairing inattention, impulsivity and hyperactive behaviour. Psychotherapy for instance is hard to comply to when ADHD is not treated first. Also compliance to medication often fails in case of untreated ADHD, leading to high relapse rates. Also the treatment of physical diseases for instance for astma, obesity and diabetes may fail in case of untreated ADHD. In fact, not treating ADHD may lead to chronicity of any other comorbid condition; one of the reasons that ADHD can be found in chronic therapy-resistent patients.

ADHD is comorbid with 34 of 35 investigated disorders and diseases in a Swedish registry study, of which many have in part a genetic background. Allergies, astma, migraine, irritable bowel syndrome, hypermobility and weak connective tissue seem to play a role in many of these diseases. Psychiatric comorbidities are also broad: anxiety, depression, bipolar disorder, autism, sleep disorders, substance abuse disorders, personality disorders, severe hormonal moodchanges across the lifespan in women, and PSTD. This summary points to the systemic nature of ADHD across the lifespan, that urgently needs more research and understanding.

The good news is that ADHD is a well treatable condition in about 80% of patients using psycho-education, stimulant and other medication, CBT and other psychotherapies. Comorbid conditions must be diagnosed and treated as well, and often first. Treatment offers stability for patients who have never experienced this from childhood. As such, the diagnosis of ADHD in adults is often a game changer, enabling patients for the first time to persevere to a better lifestyle, to prevent further damage due to sleep loss, substance abuse and obesity.

Training about diagnostic assessment and treatment is available via books, (online) training and webinars.

In this presentation, all resources will be discussed followed by Q and A.

**Disclosure of Interest:**

None Declared

